# A Case Report of a Diaphragmatic Defect Developing Into a Late-Presenting Congenital Diaphragmatic Hernia With Severe Respiratory Failure

**DOI:** 10.7759/cureus.64035

**Published:** 2024-07-07

**Authors:** Yuichi Noda, Yusuke Kusaka, Osamu Umegaki, Toshiaki Minami

**Affiliations:** 1 Anesthesiology, Osaka Medical and Pharmaceutical University, Takatsuki, JPN; 2 Intensive Care Medicine, Osaka Medical and Pharmaceutical University, Takatsuki, JPN

**Keywords:** diaphragm reconstruction, external auditory canal carcinoma, diaphragmatic relaxation, congenital diaphragmatic hernia, case report

## Abstract

Diaphragmatic hernia is a congenital malformation, often discovered in the neonatal period, and its occurrence in adults is very rare. This patient, who was completely asymptomatic until the age of 62, had developed an intestinal obstruction and went into respiratory failure after surgery for an external auditory canal carcinoma. He was subsequently diagnosed with a late-presenting congenital diaphragmatic hernia (CDH), thus requiring surgical treatment. Anesthesiologists and critical care physicians should keep in mind the possibility of CDH as well as diaphragmatic relaxation when an unexplained elevation of the diaphragm is observed perioperatively.

## Introduction

The majority of congenital diaphragmatic hernia (CDH) cases occur in the neonatal period and require surgical treatment [1-3]. However, there are rare cases that develop in adulthood after an asymptomatic period, then resulting in severe respiratory failure or intestinal obstruction [4]. This case was a late-onset diaphragmatic hernia in adulthood, with hypoxemia and abdominal pain as the initial symptoms after surgery for an external auditory canal carcinoma and required long-term mechanical ventilation and surgical reconstruction.

## Case presentation

**Figure 1:**
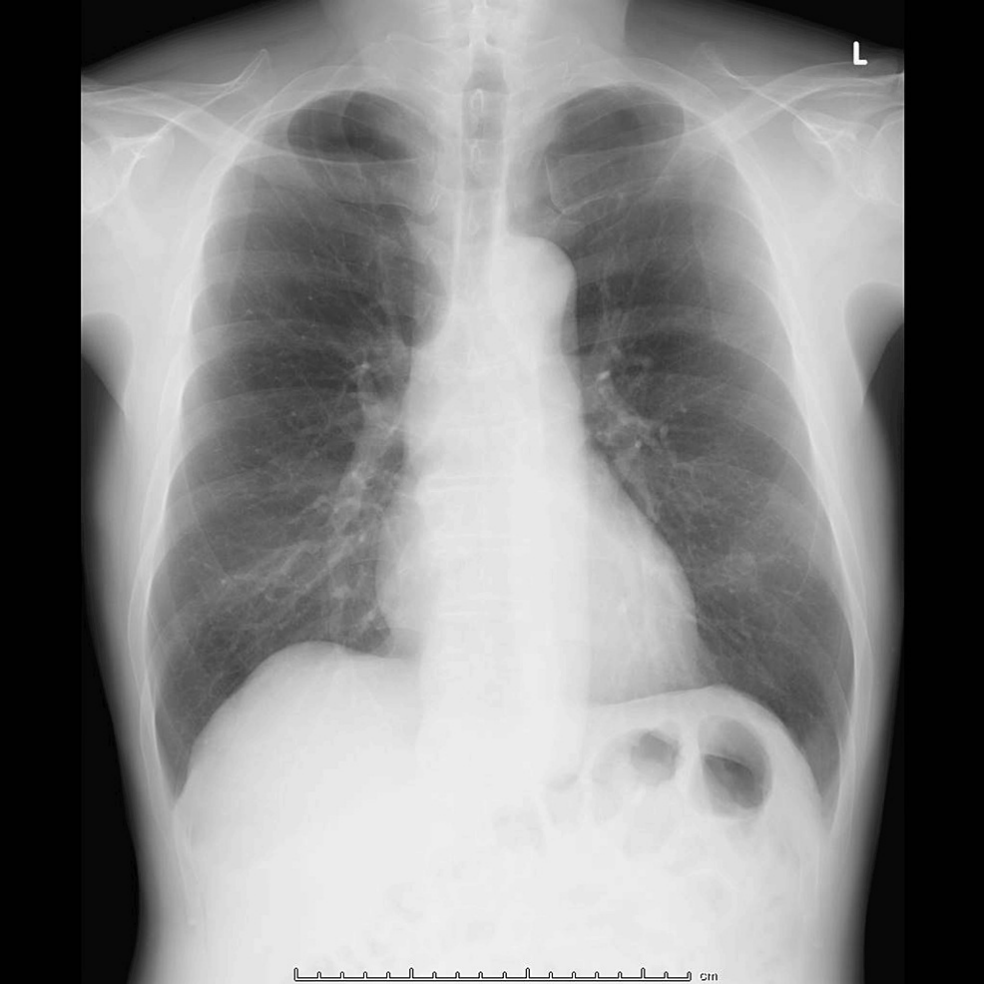
Preoperative chest X-ray. Intestinal gas was observed under the left diaphragm.

The patient is a 62-year-old male, (160 cm, 51 kg, body mass index: 19.9 kg/cm^2^), who was scheduled for the resection of a right external auditory canal carcinoma. The clinical staging was stage Ⅲ (T3N0M0), and radiation therapy was scheduled after surgery. He had a history of hypertension, was taking angiotensin receptor blockers, and had smoked 10 cigarettes a day for 42 years. He had no history of thoracic or abdominal surgery, and a preoperative chest X-ray (Figure 1) showed no abnormal findings. The patient underwent a partial lateral resection of the temporal bone and a resection of the superficial lobe of the parotid gland. After the operation, the patient was admitted to the intensive care unit (ICU) under mechanical ventilation.

**Figure 2:**
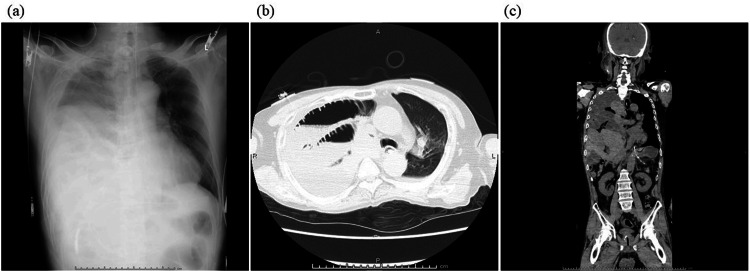
Chest X-ray and CT scan on POD 6. (a) Chest X-ray showed decreased right lung permeability. (b, c) CT scan showed that the dilated intestinal tract was fitted into the right thoracic cavity. CT, computed tomography; POD, postoperative day

**Figure 3:**
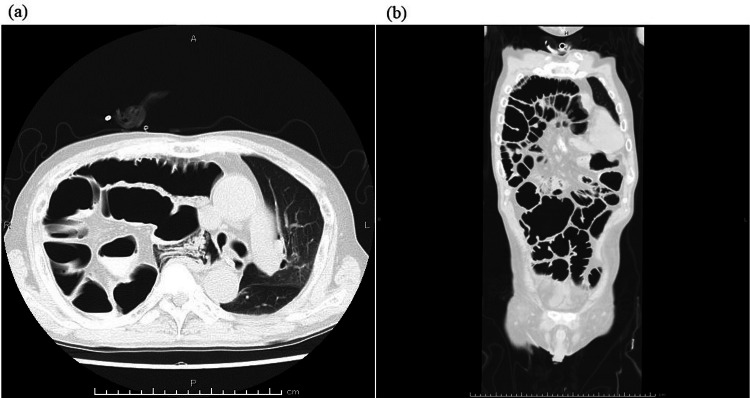
CT scan on POD 15. (a) The intestinal tract was fitted into the right thoracic cavity, resulting in a severely collapsed right lung and the mediastinum deviated to the left. (b) The right thoracic cavity was almost completely filled by the intestinal tract. CT, computed tomography; POD, postoperative day

**Figure 4:**
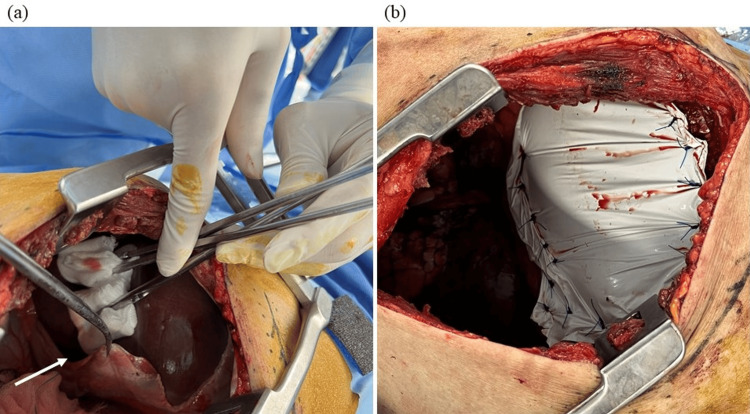
Intraoperative findings. (a) The liver was observed through the thoracic cavity, and the central tendon of the diaphragm (white arrow) could be seen. The intestinal tract was located under the gauze. No hernia sac was observed. (b) Reconstruction of the right diaphragm was performed by thoracic surgeons.

On postoperative day (POD) 1, after confirming no abnormal findings on the chest X-ray, the patient was extubated and returned to his ward. On POD 2, the patient complained of respiratory distress, and upper airway obstructive sounds were heard. Laryngoscopy revealed severely edematous epiglottis, hence an emergent tracheostomy was performed under local anesthesia. On POD 3, the patient complained of severe abdominal pain and an enhanced computed tomography (CT) scan, and an abdominal X-ray showed an elevated right diaphragm and paralytic ileus. A nasogastric tube was then inserted for gastrointestinal decompression. On POD 6, the patient showed hypoxemia, and a chest X-ray showed decreased right lung permeability (Figure 2a). A CT scan showed that the intestinal tract was fitted into the right thoracic cavity (Figures 2b, 2c), and the patient was admitted to the ICU again. Considering that respiratory failure was due to diaphragmatic relaxation, mechanical ventilation was started through the tracheostomy orifice; additionally, an ileus tube was placed for the paralytic ileus. After mechanical ventilation was started, permeability in the right lung improved within a few days. On POD 12, the ileus tube could be removed, and the patient was weaned from mechanical ventilation on POD 13. On POD 15, the patient was discharged from the ICU without any abdominal symptoms or hypoxemia. However, a chest X-ray showed elevation of the intestinal tract and diaphragm again, possibly due to the discontinuation of positive pressure ventilation. On POD 19, the patient showed hypoxemia again and was admitted to the ICU requiring mechanical ventilation. A CT scan performed before ICU admission showed that the right thoracic cavity was almost completely filled by the intestinal tract, resulting in a severely collapsed right lung (Figures 3a, 3b). On POD 26, considering that this diaphragmatic relaxation needed surgical correction in order for the patient to be weaned from mechanical ventilation, diaphragm plication was attempted by thoracic surgeons. The patient was placed in the left lateral recumbent position, and after opening the chest wall, the diaphragm was observed carefully. No hernia sac was found, and the diaphragm was defected extensively (Figures 4a, 4b). Reconstruction of the defective diaphragm was performed using a Gore-Tex sheet and without any intraoperative complications. After the operation, the patient could be weaned from mechanical ventilation on POD 27 and no longer required oxygenation by POD 30. The patient was then discharged from the ICU on POD 40 without any relapse of symptoms. However, even after diaphragm reconstruction, mild elevation of the diaphragm persisted. After chemoradiotherapy for the external auditory carcinoma, the patient was discharged on POD 59 without any complications.

## Discussion

CDH is a condition in which the abdominal visceral organs are diverted into the thoracic cavity through a vulnerable site in the diaphragm. The majority (90%) of CDH is pleuroperitoneal hernia (Bochdalek’s hernia), 85% of which occurs on the left side. Posterior sternal hernias (Morgagni or Larrey hernia) are less common, occurring in about 5% of overall CDH cases [1-3]. The incidence of CDH ranges from 1/2000 to 1/5000, with 90% occurring on the left side, 10% on the right side, and 1% bilaterally. 95% of CDH cases occur in the neonatal period, and 5% occur after infancy and are called late-presenting CDH [4]. The average age of onset of late-presenting CDH is 1.8-2.4 years, and occurrence after school age is rare. Based on these findings, the onset at 62 years of age and the right anterior diaphragmatic defect (suspected Morgani hernia) in this case were all rare. There are two modes of late-presenting CDH onset: the first mode is that due to increased abdominal pressure, intra-abdominal organs are displaced from the diaphragmatic defect that is occluded usually with a hernia sac or parenchyma. The second mode is that intra-abdominal organs migrate through the diaphragmatic defect and become trapped or twisted for some reason, thus resulting in the development of CDH [5]. In this case, the second mode is considered to be applicable because the preoperative chest X-ray did not show elevation of the right diaphragm. The prognosis of late-presenting CDH is generally good, but serious complications such as respiratory failure and gastrointestinal obstruction can occur, as in this case. CDH with respiratory failure or bowel obstruction is an absolute indication for surgery. There is still some controversy as to the preferred surgical technique. Some surgeons prefer the transabdominal approach, while others advocate the transthoracic, laparoscopic, or thoracoscopic approach. The transabdominal approach is usually preferred because of better surgical exposure and for easier hernia reduction [6]. We initially assumed that the cause of this case was diaphragmatic relaxation because the patient's symptoms temporarily improved with positive pressure ventilation. This resulted in a delayed definitive diagnosis. The main causes of acquired diaphragmatic relaxation are birth trauma, thoracic operations, and malignant tumor invasion, all of which are not applicable in this case [7]. The extensive diaphragmatic defect here induced a gigantic hernia portal and respiratory failure due to pulmonary collapse without serious complications such as gastrointestinal ischemia and perforation. This patient was completely asymptomatic until the age of 62 years; however, the hernia developed after an event such as intubation or extubation associated with general anesthesia and an emergent tracheotomy, thus making this a very rare case. In addition, even after diaphragm reconstruction, mild elevation of the diaphragm persisted, thus suggesting that the non-deficient diaphragmatic tissue was also fragile. On the other hand, one incomprehensive point in this case was the serious laryngeal edema that required an emergent tracheostomy. We considered the cause of the laryngeal edema to be the angiotensin Ⅱ receptor blocker (ARB) taken preoperatively. Angiotensin-converting enzyme inhibitors (ACEIs) and ARBs are well-known causative agents of drug-induced angioedema. The mechanism of ACEI-induced angioedema is the accumulation of bradykinin through inhibition of ACE [8]. Although ARBs themselves do not affect the renin-angiotensin-aldosterone system, they are thought to increase plasma bradykinin concentrations resulting in angioedema [9]. In addition, recurrence after their discontinuation should be noted. Beltrami et al. conducted a long-term study of 111 cases of drug-induced angioedema caused by ACEIs and reported that 46% of patients had recurrence even after discontinuation of the causative agent [8]. It is important to note that ARBs often cause recurrence more than one month after the initial onset of the disease and that they also cause delayed recurrence after discontinuation of the drug [10]. We were unable to confirm whether the patient had a history of ARB-induced angioedema in the past; however, we speculate that it could have contributed to the postoperative laryngeal edema requiring an emergent tracheostomy. The patient complained of abdominal pain and respiratory distress the day after the emergent tracheotomy, thus suggesting that the diaphragmatic hernia developed on POD2 or POD3. Severe laryngeal edema caused effortful breathing, and the intrathoracic negative pressure would have then shifted the abdominal organs into the thoracic cavity.

## Conclusions

Although late-presenting CDH in an adult is very rare, anesthesiologists and critical care physicians should keep in mind the possibility of CDH as well as diaphragmatic relaxation when unexplained elevation of the diaphragm with abdominal symptoms and hypoxemia is observed perioperatively. In addition, it is crucial to promptly consult abdominal or thoracic surgeons considering surgical treatment, if these symptoms are intractable.
